# Ecological Niche Modeling for Filoviruses: A Risk Map for Ebola and Marburg Virus Disease Outbreaks in Uganda

**DOI:** 10.1371/currents.outbreaks.07992a87522e1f229c7cb023270a2af1

**Published:** 2017-09-05

**Authors:** Luke Nyakarahuka, Samuel Ayebare, Gladys Mosomtai, Clovice Kankya, Julius Lutwama, Frank Norbert Mwiine, Eystein Skjerve

**Affiliations:** 1) Department of Food Safety and Infection Biology, Norwegian University of Life Sciences, Oslo, Norway; 2) Department of Biosecurity, Ecosystems and Veterinary Public Health, Makerere University, Kampala Uganda; 3) Department of Arbovirology, Emerging and Re-Emerging disease, Uganda Virus Research Institute, Entebbe, Uganda; Climate Change and Biodiversity Unit, Wildlife Conservation Society, Bronx, New York, United States of America; Earth Observation Unit, International Centre for Insect Physiology and Ecology, Nairobi, Kenya; Department of Biosecurity, Ecosystems and Veterinary Public Health, Makerere University, Kampala, Uganda; Department of Arbovirology, Emerging and Re-Emerging diseases, Uganda Virus Research Institute, Entebbe, Uganda; Department of Biomolecular Resources and Biolab Sciences, Makerere University, Kampala, Uganda; Department of Food Safety and Infection Biology, Norwegian University of Life Sciences, Oslo, Norway

**Keywords:** disease model

## Abstract

**Introduction:**

Uganda has reported eight outbreaks caused by filoviruses between 2000 to 2016, more than any other country in the world. We used species distribution modeling to predict where filovirus outbreaks are likely to occur in Uganda to help in epidemic preparedness and surveillance.

**Methods:**

The MaxEnt software, a machine learning modeling approach that uses presence-only data was used to establish filovirus – environmental relationships. Presence-only data for filovirus outbreaks were collected from the field and online sources. Environmental covariates from Africlim that have been downscaled to a nominal resolution of 1km x 1km were used. The final model gave the relative probability of the presence of filoviruses in the study area obtained from an average of 100 bootstrap runs. Model evaluation was carried out using Receiver Operating Characteristic (ROC) plots. Maps were created using ArcGIS 10.3 mapping software.

**Results:**

We showed that bats as potential reservoirs of filoviruses are distributed all over Uganda. Potential outbreak areas for Ebola and Marburg virus disease were predicted in West, Southwest and Central parts of Uganda, which corresponds to bat distribution and previous filovirus outbreaks areas. Additionally, the models predicted the Eastern Uganda region and other areas that have not reported outbreaks before to be potential outbreak hotspots. Rainfall variables were the most important in influencing model prediction compared to temperature variables.

**Conclusions:**

Despite the limitations in the prediction model due to lack of adequate sample records for outbreaks, especially for the Marburg cases, the models provided risk maps to the Uganda surveillance system on filovirus outbreaks. The risk maps will aid in identifying areas to focus the filovirus surveillance for early detection and responses hence curtailing a pandemic. The results from this study also confirm previous findings that suggest that filoviruses are mainly limited by the amount of rainfall received in an area.

## Introduction

Uganda has experienced eight filovirus outbreaks; five Ebola Virus Disease (EVD) and three Marburg virus disease (MVD), between 2000 and 2016, more than any other country in the world.

The first outbreak in Uganda was caused by Ebolavirus of the species Sudan ebolavirus in 2000 in the Northern district of Gulu, where 425 cases were registered with a case fatality rate (CFR) of 53%^1^. The second outbreak was caused by Bundibugyo Ebolavirus in the western part of Uganda bordering with Democratic Republic of Congo (DRC), with 192 cases and a CFR of 34%^2^^,^^3^ . In 2011, another EVD outbreak occurred where only one case was involved in Luweero district Zirobwe village, 45 km North of Uganda’s Capital City Kampala^4^. Two more EVD outbreaks were observed in 2012, one in June in the Western District of Kibale and another in November, Luweero district in Central Uganda^5^.

Likewise, three outbreaks of MVD have occurred in Uganda; the first one was in Kamwenge district in 2007 associated with mining activity in the Kitaka gold mine that is occupied by bats^6^. This outbreak was later linked to cave-dwelling Egyptian fruit bats (*Rousettus aegyptiacus*) that occupy these mines, as they tested positive for Marburg virus by polymerase chain reaction (PCR)^7^^,^^8^. Another outbreak of MVD was in 2012 where several districts were involved with a CFR of 58% (15/26)^9^. This outbreak was also traced back to the same gold mines in Western Uganda, and subsequent testing of the bats in the mines revealed a spill over to human populations^10^. The latest MVD outbreak was in Kampala where the only fatal case was a health worker, and no other cases were identified^11^.

It is hypothesized that distribution of filoviruses is limited by the distribution of the bats, which are known probable reservoirs. All the filovirus outbreaks in humans have been reported to originate from Sub-Sahara Africa and only one species, Reston virus that is not known to infect humans was detected outside Sub-Sahara Africa in The Philippines^12^. It has been suggested that transmission from the natural reservoir occurs when humans get into contact with the reservoir or its body fluids such as feces, urine, and blood via activities such as hunting and consumption of bush meat^13^. Because previous outbreaks in Central Africa have been linked to reports of bush meat consumptions and deaths of wildlife^14^, many hypotheses have been put forward to suggest wildlife such as bats, primates, and antelopes as possible sources of infection. The debate on bats as potential reservoirs of Ebolaviruses is still not concluded, as no *Ebolavirus* has been isolated from bats despite finding some bats seropositive for Ebolavirus and others with viral RNA^15^. The role of non-human primates as reservoirs has been unconvincing since they do die from infection with filoviruses^16^^,^^17^^,^^18^^,^^19^. Other wildlife that has been reported to be infected by *Ebolavirus* was one duiker, whose bone tested positive by PCR in Republic of Congo bordering Gabon^19^. Dogs and pigs are the only domestic animals associated with ebolaviruses. Dogs were found to be IgG seropositive in Gabon^20^ whereas Reston virus has been reported in pigs and have shown potential for infection with Ebola virus^21^^,^^22^^,^^23^. Unlike EVD, there is progress in research in trying to describe the reservoirs of Marburg virus. Bats of species *Rousettus aegyptiacus*, found in Kitaka gold mine and Python cave from the Albertine region in Western Uganda have been described as potential reservoirs of Marburg virus in Uganda^8^^,^^10^^,^^24^. The bats in these caves have been linked to three MVD outbreaks, where artisanal gold miners got infected with Marburg virus^6^^,^^9^. Transmission of Marburg virus in human populations just like Ebolaviruses happens after a spillover event from the natural reservoir in wildlife. Lack of a clear reservoir and true source of infection or spill-overs into human populations has been a call for alternative methods of heightening surveillance and developing risk maps is one of them.

Situated in the rich and complex ecological systems with high biodiversity in East Africa, Uganda is not only affected directly by filovirus outbreaks but also vulnerable to outbreaks from neighboring countries such as DRC. For epidemic preparedness and response, Uganda’s health surveillance system needs to know where and when these epidemics are likely to occur. This will allow them to conduct active surveillance focusing in those areas for early detection to avoid pandemics and also focus research on reservoirs. This can be achieved by applying spatial epidemiology modeling techniques. One such technique is Ecological Niche Modeling (ENM) also known as Species Distribution Modeling (SDM), that has been used to establish the relationship between species and their environment^25^^,^^26^^,^^27^^,^^28^. ENM has also been used to predict the ecology and distribution of filoviruses before. Peterson *et al* (2014) used a Genetic Algorithm for Rule-Set Production (GARP) model to predict suitable environments for filoviruses as being in afro-tropics where EVD was being predicted more in the humid rain forest of Central and West Africa while MVD was more predicted to occur in the drier and more open areas of Central and East Africa^29^. More efforts were made to improve the spatial prediction model for MVD for Africa using a Bioclimatic variable (Bioclim)^30^, which predicted filoviruses mainly in Zimbabwe and abroad potential distribution across the arid woodland regions of Africa^31^. Furthermore, Pigott *et al* (2014) developed zoonotic niche maps for Marburg and Ebola viruses in Africa using species distribution models^32^^,^^33^. In these maps, they have predicted EVD at risk areas occupied by 22 million people while MVD is predicted to occur in 27 countries across Sub-Sahara Africa. Enhanced vegetation index which corresponds to high levels of rainfall was identified as the most important variable limiting the distribution of the Ebola virus in Africa^32^^,^^33^.

These predictions are not country specific, and they lack details of individual countries regarding vector and raster data. For example, they used online databases that are not accurate especially in estimating environmental covariates and getting coordinates of index cases, hence, affected countries find these maps limited for focused and targeted surveillance

A Maximum Entropy species distribution modeling environment (MaxEnt) has been used to predict the ecological niche for various species. The MaxEnt algorithm uses presence-only occurrence records to estimate the actual or potential geographic distribution of a species^34^ and has been known to outperform other species’ distribution modeling approaches such as Domain, Generalized Additive Models (GAM), Generalized Linear Models, Genetic Algorithm (GARP) and Bioclim^35^.

MaxEnt models have been used widely to predict ecological niches of different vectors and disease-causing organisms^36^^,^^37^^,^^38^^,^^39^^,^^40^^,^^41^^,^^42^^,^^43^, but it has not been used for prediction of filovirus outbreaks in Uganda. Briefly, MaxEnt is a multipurpose machine-learning technique and aims at estimating the probability of distribution of a species occurrence using the environmental features. Our major aim was to develop a country-specific risk map for Uganda using updated data on EVD/ MVD outbreaks and bat occurrence and environmental variables specific for Uganda using the MaxEnt modeling approach. The model outputs will improve filovirus epidemic preparedness, surveillance and response, and in the search for a reservoir especially in a disease prone country like Uganda

## Materials and methods

**EVD, MVD and Bat occurrence data**

A total of 16 locations of the Ebolavirus outbreaks in Uganda since 2000 was obtained from published databases^44^. An additional 27 occurrence points for Ebola and Marburg virus diseases outbreaks were collected from the field where these outbreaks occurred especially for new outbreaks whose locations were not collected before. All locations where confirmed cases of Marburg or Ebola viruses were reported were collected with Global Positioning System (GPS) receiver and points were entered into an Excel spreadsheet. A total of 43 filovirus outbreak occurrence points (30 for EVD outbreak and 13 for MVD outbreak) were used for this prediction model (Supporting Information S1 File; see Appendix). These filovirus occurrence points represent households in villages where confirmed cases were residing. Due to the contagious nature of filoviruses, one household had more than one cases hence the reason for not using all the 562 EVD cases and 20 MVD cases. A fruit bat location survey was also done to determine the location of fruit bats in a cross-section of Uganda. We purposively selected districts to scout for bats based on previous filovirus outbreaks and anecdotal reports of bats in trees. Using a snowballing approach, we collected 84 fruit bat locations using a GPS receiver from different districts of the country. Here community members acted as informers of the roosting locations of fruit bats and caves that contain bats.

An additional, 517 bat locations from all over Uganda were generously provided by Kityo Robert (Department of Zoology, Makerere University Kampala Uganda) also published in Uganda Bat Atlas^45^, resulting in a total of 601 bat coordinates (Supporting Information S1 File; see Appendix).

**Environmental covariates**

Ecologically suitable environmental covariates for filovirus outbreaks for Uganda were compiled from Africlim^46^, with a spatial resolution of 1 km. The environmental covariates considered were moisture (mean annual rainfall, rainfall wettest month, rainfall driest month, rainfall seasonality, rainfall wettest quarter, rainfall driest quarter, annual moisture index, moisture index arid quarter, number of dry months, length of longest dry season) and temperature variables (mean annual temperature, mean diurnal range in temperature, isothermality, temperature seasonality, maximum temperature warmest month, minimum temperature coolest month, annual temperature range, mean temperature warmest quarter, mean temperature coolest quarter, potential evapotranspiration). We used ENMTOOLs; a toolbox that facilitates quantitative comparisons of environmental niche models^47^ to test for multicollinearity between the predictor variables and we ran a pairwise Pearson correlation, and only variables with less than (+/-0.75) correlation were retained in the final prediction model (Supporting Information S2 File; see Appendix). After this test, only seven environmental variables were retained (Table 1); three moisture variables (Rainfall seasonality, Rainfall driest quarter, and mean annual rainfall) and four temperature variables (Temperature seasonality, Mean diurnal range in temperature, mean annual temperature and Isothermality).

**Ecological Niche Model**

We used MaxEnt Version 3.3k for modeling distribution of filovirus using default settings (Auto features, convergence threshold=0.00001, the maximum number of background points=10,000, regularization multiplier=1). A logistic probability map was generated showing the relative probability of the presence of filoviruses survival on a scale ranging between 0 and 1^48^. The occurrence data was subdivided into k-folds where 25% was set aside for testing the accuracy of the model, whereas 75% was used for training the model. However, there were few presence records (10) for the Marburg cases therefore, all the records were used in training the model. The Receiver Operating Curve (ROC) was used to assess the overall model predictive performance, a measure of the ability of the model to distinguish presence from absence of a species with a value of 1 indicating a perfect prediction while 0.5 is as good as a random prediction^49^^,^^50^. A jackknife test was used to evaluate individual covariate importance in the model developments (Supporting Information S3 File; see Appendix). To improve model robustness, 100 replicates were averaged for the final model outputs. MaxEnt outputs were imported into ArcGIS 10.3 mapping software to develop final maps.

**Figure:**
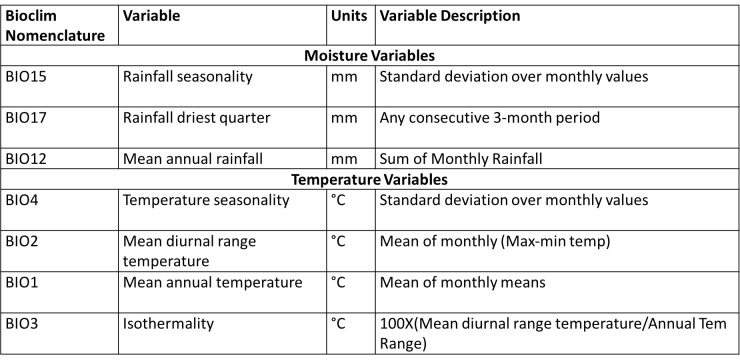
**Table 1:** Environmental variables used in the models

## Results

**The bat occurrence and filovirus outbreak locations**

As shown in Figure 1, bats are distributed all over Uganda, with a high distribution around water bodies which is a core need for survival. Areas around Lake Victoria, River Nile, and Western Rift Valley have high numbers of bats. Their locality is in line with regions that have reported filovirus outbreaks in Uganda.

**Map of Uganda showing outbreak locations of Ebola and Marburg virus diseases and bat locations included in the Maxent modeling Environment:**
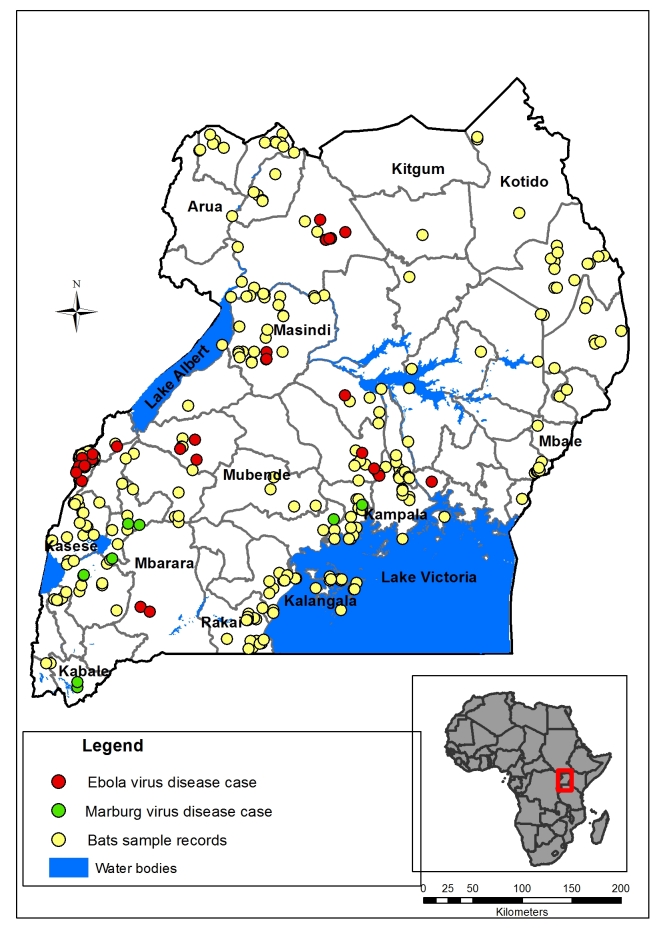

**Bat distribution in Uganda**

From 100 bootstrap replicates, a bat distribution map was generated (mean AUC=0.80; SD=0.012). Compared to a random prediction of AUC 0.5, our model was able to distinguish presence from the absence of bats within the geographic space with a high accuracy^51^. The relative probability of presence (RPP) ranged from highly suitable areas represented by red to orange colors to unsuitable areas represented by the green color in Figure 2A. The map shows that most areas in Uganda are suitable habitats for bats (both insect and fruit bats) with high RPP occurring in the following districts; Mbarara, Bushenyi, Bundibugyo and Kabale located in the western part of Uganda, around Lake Victoria (Kampala and Luweero districts) and in eastern region of Mbale and Soroti districts. Moderately suitable regions largely cover most parts of Uganda. The RPP of bats were mainly influenced by rainfall driest quarter with 24.7%, mean annual rainfall with 17.2%, mean diurnal range in temperature with 14.5%, and isothermality with 11.5% (Table 2).

**Ebola virus distribution**

High RPP for EVD outbreak was predicted in more than half of the country with hotspots in Western Rift valley districts of Bundibugyo, Masindi, Kibale and Hoima, Kasese, Kabarole, Kamwenge, Bushenyi and Ibanda as shown in Figure 2B (mean AUC=0.90; SD=0.024). In Central Uganda, Luweero, Kayunga, Mpigi, Kampala, Mityana and Nakasongola districts are predicted as potential areas for EVD outbreaks. In the eastern part of the country, it is mainly the Busoga region along River Nile and Mbale district around Mt. Elgon that are potential EVD hot spots. Other places that have not recorded outbreaks before but are predicted as potential probable areas for the spread of EVD include areas surrounding Lake Victoria and around Mount Elgon. A low RPP for EVD outbreak was predicted in North Eastern Uganda (Karamoja region) and Northern Uganda in the districts of Kitgum and Pader. Rainfall seasonality (33.2%), Mean annual rainfall (22.7%), rainfall of the driest quarter(20.8%) and mean diurnal range in Temperature (9.9%) had the highest relative contribution in predicting Ebola virus ecological suitability (Table 2).

**Marburg virus disribution**

The map in Figure 2C shows that Western, Southwestern and Central Uganda are potential areas for outbreaks of Marburg cases(AUC=0.92). Unlike predicted potential areas for EVD, predicted areas for MVD are mainly in the western sub-regions of Ankole, Tooro, Bunyoro, and Rwenzori region extending into DRC. Areas in the North and Eastern part of Uganda have a low or no relative probability of presence for MVD outbreaks as shown by the green color in Figure 2C. Temperature seasonality (68.2%) and rainfall seasonality (25.3%) contributed heavily to the model prediction (Table 2). Notably, temperature seasonality had the highest influence in MVD model compared to other variable contributions in all the models. However, the occurrence points were few in number to give us an accurate prediction.

**Maps showing bats, EVD and MVD distribution in Uganda with high Relative Probability Presence represented in red while low in green.**
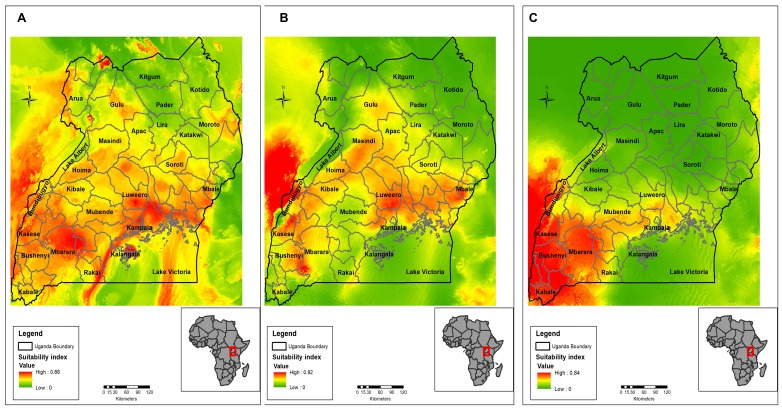
A: Relative probability of presence of bats, hypothesized as reservoirs of filoviruses (AUC=0.80), B: Relative probability of presence of Ebola Virus disease outbreak (AUC=0.90), C: Relative probability of presence of Marburg Virus disease outbreak (AUC=0.92.

**Filovirus distribution**

Combining Marburg and Ebola virus occurrence points (Figure 3), we see the range of the possible distribution of filovirus, mainly in western, southwestern Uganda, Victoria basin districts and eastern Uganda (mean AUC=0.90; SD =0.023). Predictor variables that contributed more than 75% in the model include; rainfall seasonality (29.6%), rainfall of the driest quarter (26.3%), Temperature seasonality and mean annual rainfall (14.9%) (Table 2).

**Map showing areas of the relative probability of the presence of filovirus (Ebola and Marburg virus) outbreak in Uganda.**
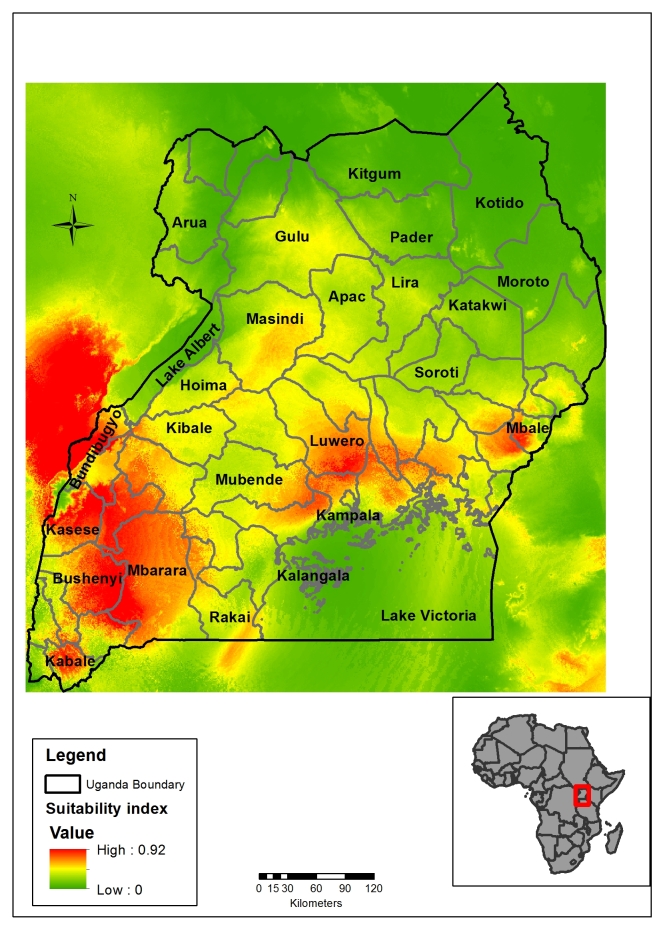
(AUC=0.9)

**Variable Contribution to the prediction models**

Figure 4, shows the response curve of the most important variable for each of the models (The response curves of all the predictor variables in all the four models are in Supporting Information S4 File; see Appendix). The response curves show the mean response of the 100 replicate MaxEnt runs (red) and the mean +/- one standard deviation. Figure 4Asuggests that probability of bats occurrence are optimal at 30 – 40 degree Celcius during the driest quarter(Bio17). MVD occurs in areas where temperature variability (Bio4) is minimal (Figure 4C) whereas EVD (Figure 4B) and both the filovirus (Figure 4D) occurs in areas with minimal rainfall variability (Bio15).Bio4 and Bio15 show how temperature and rainfall vary over a given year based on standard deviation. The response curves, show that MVD occurs in areas with low variability of temperature and EVD / Filoviruses occur in areas with low variability of rainfall. Bio4 contributes 68% to the relative probability of occurrence of MVD, which indicates that MVD is limited when there is high variability in temperature across the year. Rainfall variables contributed about 75% to the to the relative probability of occurrence of EVD. The results indicate that EVD is limited by the amount of rainfall received in an area. Higher rainfall increases the relative probability of occurrence of EVD.

**Figure:**
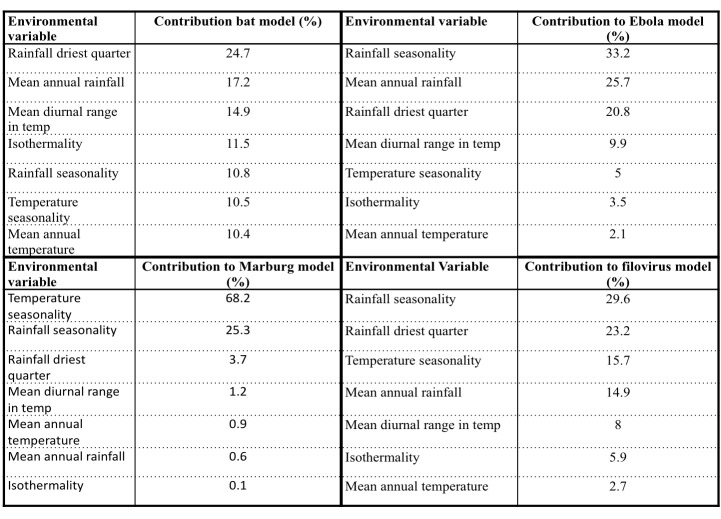
**Table 2:** Environmental variable contribution in the MaxEnt prediction models

**Response curves of environmental variables that contribute highest to each of the prediction models.**
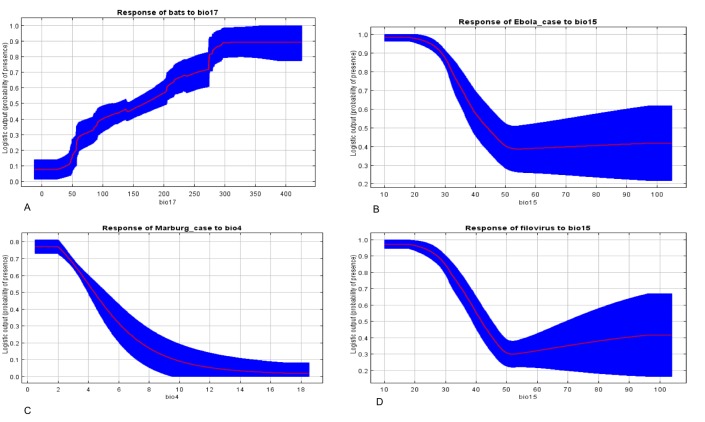
A: Rainfall driest quarter(BIO17) vs Relative probability of bat presence. B: Rainfall seasonality(BIO15) vs. Relative probability of presence of Ebola virus outbreak; C: Temperature seasonality(BIO4) vs. Relative probability of presence of Marburg virus outbreak; D: Rainfall seasonality(BIO15) vs Relative probability of presence of Ebola or Marburg virus disease outbreak

## Discussion

We used seven environmental variables in this model prediction. This was after assessing for collinearity in the model and removing all the collinear variables. Variable contribution assessment as shown inTable 2 showed that rainfall variables were the most important predictors. The importance of rainfall or precipitation and moderate to high temperature was highlighted by Peterson *et al* (2004) when they modeled filovirus distribution in Africa using GARP model^29^^,^^31^. Rainfall is important for the obvious reason that it provides water which is very important for bats survival^52^^,^^53^. Rainfall also provides for the development of fruiting trees that provide roosting areas for bats as well as food for fruit bats. Uganda is endowed with many water bodies and several rainforests, and hence bat distribution tends to be all over the country as seen in Figure 2A. Bats are hypothesized to be reservoirs for filoviruses; their distribution tends to correlate with that of filovirus predicted niches (Figure 3). Although we have some progress with Marburg virus in trying to describe bats as a source of infection for humans^7^^,^^8^^,^^10^^,^^54^, more research needs to be done especially on the reservoir for Ebola virus as these models can only give a clue as to the possible surveillance sites and possible areas to focus the research and to identify other potential reservoirs for filovirus. Temperature and rainfall seasonality were the most important environmental variables contributing to spatial prediction model for the Ebola and Marburg viruses. Seasonality has been found to be key in outbreaks of filoviruses, especially MVD as was reported in an ecological study by Amman et al. 2012^8^. In this study, outbreaks of MVD are associated with the birthing seasons of adult juvenile bats when the virus circulation was high. This is further validated by a high percentage contribution (68.2%) of temperature seasonality into the MVD outbreak prediction model (Table 2). The relative probability of the presence of a Marburg outbreak is higher (80%) and at very low-temperature seasonality, which is a standard deviation (SD) over monthly values (Figure 4C). Therefore, areas with fewer variations in monthly temperature and rainfall are more likely to experience MVD and EVD outbreaks and this has been predicted by the models in Figures 2 & 3. The areas shown on the risk maps with a high relative probability of the presence of an outbreak are mainly in the South, the West and Central Uganda that have minimal temperature and rainfall variations compared to North Eastern Uganda that is not predicted for filovirus outbreaks except for bat presence. Bat presence model is mainly influenced by the variable rainfall driest quarter (24.7%) and mean annual rainfall (17.2%) (Table 2). As these variables increase, the relative probability of the presence of bats tends to increase. Areas of high rainfall are more likely to be forested or with many fruiting trees that provide a suitable habitat for bats, and this is true for three-quarters (75%) of Uganda.

Whereas Pigott *et al* (2015) used environmental covariates with a spatial resolution of 5km in their models^55^^,^^56^ , we used Africlim data with 1km spatial resolution. High-resolution data increases the accuracy of the models, and this was observed in our study by a high AUC greater than 0.8 recorded in all models.

The predictions show that a big part of Uganda, a country of 34 million people is at risk of a filovirus outbreak. This is more so in the Lake Victoria basin districts and in the Albertine Rift region districts and the areas that occur in between (Figure 2 & 3). The Albertine Rift region provides a variety of habitats characteristic of the East African savannahs and the West African rain forests that are suitable for reservoirs of filoviruses. According to Uganda National Meteorological department, these are the areas that receive near or above normal seasonal rainfall, and seasonal temperature variations are minimal^57^. Moreover, we see from variable contribution (Table 2), response curves (Figure 4x) and Jackknife test (Supporting Information S3 File; see Appendix) that rainfall and temperature seasonality were the most important variables in predicting outbreaks. The lower the variability in rainfall and temperature, the higher the relative probability of presence and vice versa and an increase in mean rainfall variables increases relative probability of having a filovirus outbreak (Figure 4). Indeed, six filovirus outbreaks have happened in this region, one caused by Bundibugyo ebolavirus in Bundibugyo district in the plains of Rwenzori mountains^2^, Sudan Ebolavirus in Kibale district^5^ and four outbreaks of Marburg virus all linked to Python cave and Kitaka gold mines in Kamwenge, Ibanda, and Rubirizi districts^6^^,^^9^^,^^58^^,^^59^. This remains a high-risk area with cross-border movement between Uganda and DRC where another EVD outbreak happened in 2012 in the neighboring Isiro region^60^ The Albertine Rift of East Africa needs to remain under heightened surveillance especially now that oil exploration will be taking place bringing an invasion of virgin lands by humans and interaction of wildlife and humans. Important to note also in this region has six national parks of Uganda (Queen Elizabeth National Park, Murchison Falls National Park, Kibale Forest National Park, Semiliki National Park, Bwindi Impenetrable National Park and Mgahinga National Park) on Uganda side and several other national parks on the DRC and Rwanda side as well as several forest reserves all of which harbor various species of bats and other possible reservoirs of filoviruses. All outbreaks of Marburg virus disease in Uganda have been investigated, and all originate from the old gold mines found in Ibanda and Kamwenge district^6^^,^^9^ in the Western Rift Valley which validates MVD distribution model in Figure 2C as it shows these as high-risk areas for filovirus outbreaks. A similar finding was obtained by Peterson and Samy 2016 in a recent model using MaxEnt as they predicted Sudan Ebola virus to occur in North Western Uganda between Lake Albert and Lake Vitoria^61^. We also see areas that have not had EVD outbreaks before such as West Nile region being predicted potential areas for EVD outbreak. These include areas along River Nile and areas bordering South Sudan and DRC Figure 2B). From Table 2, we see that rainfall variable contribute a higher percentage of the relative probability of presence for filovirus habitants. These areas receive average annual rainfall between 100-120mm and are endowed with high vegetation cover and water bodies all of which make the region conducive for reservoirs of filoviruses

Another area of high concern predicted by this model is Lake Victoria basin and districts in Nile River basin in Central districts of Uganda. Uganda has reported three outbreaks of filoviruses previously detected in these regions in the districts of Luweero^4^^,^^5^ and Mpigi^11^^,^^62^. This also can be attributed to the variety of habitats provided by water bodies, forests, swamps and high presence of fruit bats and other wildlife in this region. For example, the Kasokero cave that is the habitat of many Egyptian fruit bats that are known to harbor Marburg virus is found just on the banks of Lake Victoria in Masaka district, and several pathogens have been isolated from this cave 63. This is at the same time a highly-populated region with Uganda’s capital in the middle and needs to be heightened surveillance. We also predicted other regions that have not heard outbreaks of filoviruses in the past such as the Eastern region of Mbale, Busia and Tororo districts near the Mt. Elgon regions bordering with Kenya. This also still attributed to by the presence of suitable conditions for survival of putative reservoirs of Ebola and Marburg viruses. An outbreak happened in neighboring Kenya in Kitum cave^64^^,^^65^. These newly detected hotspots need to be kept under surveillance for early outbreak detection and response.

**Limitations**

We build on filovirus risk mapping efforts by Pigott et al^32^^,^^33^^,^^56^ and Peterson et al^29^^,^^31^^,^^61^ all of which have been done at the continental level of Africa. Their work was more ecologically oriented and more focused on identifying the ecological niche of species, they lacked country specific details that we bring in this publication with a bias in public health surveillance and outbreak detection rather that ecological niche identification. For public health surveillance of a country like Uganda, all filovirus species (Marburg virus, 5 Ebola virus species) are of public health importance. This makes our models more sensitive as opposed to specific risk map and hence more useful tools to the surveillance activities. There is already enough evidence of filovirus outbreaks in Uganda, especially areas predicted by our models. Focused surveillance needs to be done in these areas and bring additional surveillance in other new predicted areas where we have not heard outbreaks before. So we think modeling the map at a genus level (filovirus) level as opposed to species level is more informative for surveillance but may not be the best for ecological studies for which is not the purpose of this study. We know that disease outbreak is a combination of very many factors, not only suitable environmental covariates. However, we were not able to include as many factors as possible in this model because of lack of or poor quality data for Uganda specifically. We did not use bats as a predictor in our model because of their widespread distribution all over Uganda, otherwise doing this would lead to misleading interpretation and bias of potential outbreak hotspots as being the whole country. Another point would have been good to include in the prediction model are socio-economic factors since they play a big role in the outbreak of filoviruses.

**Conclusion**

Ecological niche modeling techniques have been widely used in predicting where disease outbreaks are likely to occur, more specifically where species have suitable living conditions depending on their environmental factors. The MaxEnt modeling algorithm uses presence only occurrence data and has been useful to estimate species’ niche in environmental space where absence records for a species are not available as it is the case with filoviruses. Given the public and global importance of filoviruses, developing models that predict where they are likely to occur is very important, and efforts in this direction have been done focusing on the African continent. In this paper, however, we focus on Uganda as one of the affected countries; and develop a country-specific prediction map. We show which places in Uganda that are hot spots for filovirus disease outbreaks and hence a focus on surveillance for early detection. Until now, no verified true reservoir for Ebola virus has been identified, and studies in this direction are still ongoing. In the absence of a known reservoir, these risk maps will help in early focused surveillance and early detection to avoid a global catastrophe like it happened in West Africa in 2014. Minimal seasonal variations in temperature and rainfall were important predictors of a filovirus outbreak. We believe these risk maps will be important in targeted surveillance, research and epidemic preparedness for Uganda. The results from this study also confirm previous findings that suggest that Filoviruses are mainly limited by the amount of rainfall received in an area.

## Appendix

**Supporting Information**

S1 File: https://doi.org/10.6084/m9.figshare.5306875

S2 File: https://doi.org/10.6084/m9.figshare.5306908

S3 File: https://doi.org/10.6084/m9.figshare.5306914

S4 File: https://doi.org/10.6084/m9.figshare.5306932

## Corresponding Author

Dr. Luke Nyakarahuka: n3luke@covab.mak.ac.ug; nyakarahuka@gmail.com

## Competing Interests

The authors have declared that no competing interests exist.

## Data Availability

All data is available in the paper and supporting files which can be found on figshare as follows: S1 File: Occurrence dataset used (Filovirus and Bats Occurrence coordinates) (10.6084/m9.figshare.5306875 <https://doi.org/10.6084/m9.figshare.5306875>)**; **S2 File: Results of the quantitative comparisons of environmental variables to test for multicollinearity (10.6084/m9.figshare.5306908 <https://doi.org/10.6084/m9.figshare.5306908>); S3 File: A jackknife test result to evaluate individual covariate importance in the model developments (10.6084/m9.figshare.5306914 <https://doi.org/10.6084/m9.figshare.5306914>); S4 File: The response curves of all the predictor variables in all the four models (10.6084/m9.figshare.5306932 <https://doi.org/10.6084/m9.figshare.5306932>).
